# Candidates Cell Sources to Regenerate Alveolar Bone from Oral Tissue

**DOI:** 10.1155/2012/857192

**Published:** 2012-02-14

**Authors:** Masahiro Nishimura, Kazuma Takase, Fumio Suehiro, Hiroshi Murata

**Affiliations:** Department of Prosthetic Dentistry, Graduate School of Biomedical Sciences, Nagasaki University, Sakamoto 1-7-1, Nagasaki 852-8588, Japan

## Abstract

Most of the cases of dental implant surgery, especially the bone defect extensively, are essential for alveolar ridge augmentation. As known as cell therapy exerts valuable effects on bone regeneration, numerous reports using various cells from body to regenerate bone have been published, including clinical reports. Mesenchymal cells that have osteogenic activity and have potential to be harvested from intra oral site might be a candidate cells to regenerate alveolar bone, even dentists have not been harvested the cells outside of mouth. This paper presents a summary of somatic cells in edentulous tissues which could subserve alveolar bone regeneration. The candidate tissues that might have differentiation potential as mesenchymal cells for bone regeneration are alveolar bone chip, bone marrow from alveolar bone, periosteal tissue, and gingival tissue. Understanding their phenotype consecutively will provide a rational approach for alveolar ridge augmentation.

## 1. Introduction

For increasing the success rate of implant surgery, various scaffolds and methods have been developed to augment atrophic alveolar ridge. Generally, autologous bone augmentation has been penetrating as a golden standard bone augmentation; however, most of the patients might not be feasible for extracting their own bone, just because it is a healthy part. To avoid aforementioned high-invasive treatments, cell therapy has recently been researched in this age of rapid advance.

**Figure 1:**
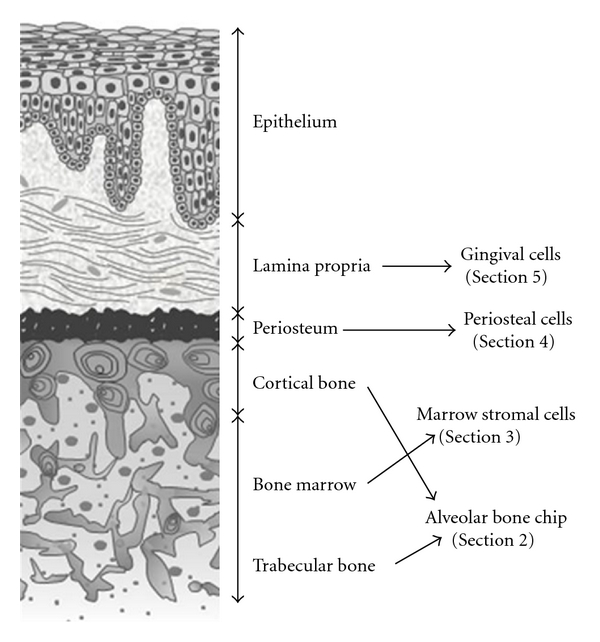
Sectional scheme of edentulous alveolar ridge. Figure shows the origin of candidate tissues and the cells we could harvest from alveolar bone chip, bone marrow, periosteum, and gingiva.

Combination of mesenchymal cells and ceramic scaffold for bone regeneration has been documented [1]. Cultured mesenchymal cells introduced into ceramic scaffolds exhibits robust osteogenic potential, with bone forming into pore regions of scaffolds. After this report, numerous reports using various cells to regenerate bone and sophisticate reviews for bone regeneration of craniofacial site have been published [2–7]. Usage of tooth, including periodontal ligament or pulp, has also been reported that multipotential stromal cells which are composed above mentioned were exploited in bone or periodontal regeneration [8, 9]. Although bone augmentation is mostly fundamental to elderly, they unfortunately follow to edentulous patients in aging society. Thus, this paper focuses on adult mesenchymal cells that could be able to expand from edentulous jaw.  Figure 1 shows the tissues we describe in this paper by sectional scheme of edentulous alveolar ridge.

## 2. Alveolar Bone Chips

Osteoblasts-like cells migrated from alveolar bone chips have generally high osteogenic activity. Essentially, mammalian bones are in the form of two different ways: long bones via endochondral ossification and flat bones via intramembranous ossification. Orofacial bone is mainly formed via intramembranous ossification, and a part of mandibular is formed via endochondral ossification. These bony types show considerable differences in protein composition [10]. Harvesting bony chips from various sites implicate that origins of the osteoblastic cells (from maxilla or mandibular, from cortical or trabecular bone) are distinct from each reports; furthermore osteogenic activity, expression of surface antigens, or ability for ectopic bone formations might be different among each report, beside cell isolation protocols are different among each report. Majority of culture protocol of osteogenic cells from alveolar bone are wash bone specimens in PBS, scrape to remove attached soft tissue and periosteum, brake into small pieces, and wash with collagenase (1 to 2 mg/mL) dissolved in culture medium [11, 12]. In some reports, osteogenic cells were collected without collagenase [13–15]. However, despite harvesting bony chips from healthy site is essential when we use these in clinic, it is not feasible for all patients just because of the invasive operation. In addition, it is still not clear how amount of bony chips is enough to regenerate each part of alveolar ridge and which part of bone cells are suitable to keep augmented bone volume on long prognosis. 

## 3. Bone Marrow from Alveolar Bone

The reason why iliac crest bone marrow is the most documented bone marrow transplantation is because they have been corrected for bone marrow transplantation in clinic as usual. Bone marrow stromal cells (BMSCs) have been reported their ability of multipotent differentiation to bone, cartilage, tendon, muscle, adipose tissue, and neuronal tissue [16–18]. Bone regenerative clinical studies using BMSCs, collected from iliac crest to reconstruct jaw defects, have been reported [19, 20]. Kawaguchi et al. reported that iliac crest BMSCs enhance periodontal tissue regeneration as well [21, 22]. Alveolar BMSCs, however, is essentially different from axial BMSCs from their differential potential or their gene expression pattern [23, 24]. Embryologically, alveolar tissues including alveolar bone marrow are originated from neural crest cells, but other bone marrows are from mesoderm [25, 26]. Cherubism [27], Treacher Collins syndrome [28], craniofacial fibrous dysplasia [29], and hyperparathyroid jaw tumor syndrome [30] affect only jaw bones, indicating that orofacial bone development differs from that of axial and appendicular bone formation. Whitaker's group have reported that membranous bone underwent less resorption than endochondral bone in monkey model [31], and they found the rapid vascularization on membranous onlay bone grafts in rabbit model [32]. In human alveolar cleft defects, chin bone was better incorporated, significantly less resorbed than iliac crest bone [33, 34]. In histomorphometry, autologous grafts obtained from calvarial sources for sinus lift procedure present a significantly higher degree of bone volume in contrast to bone harvested from the iliac crest [35]. In *in vitro* and *in vivo* study, Akintoye et al. have investigated skeletal site-specific phenotypic and functional differences between orofacial (maxilla and mandible) and axial (iliac crest) human BMSCs. Compared with iliac crest cells, orofacial BMSCs proliferated more rapidly with delayed senescence, expressed higher levels of alkaline phosphatase, and demonstrated more calcium accumulation *in vitro*. Orofacial BMSCs formed more bone *in vivo*, while iliac crest BMSCs formed more compacted bone that included hematopoietic tissue and were more responsive *in vitro* and *in vivo* to osteogenic and adipogenic inductions [36]. Comparing with the osteogenic properties of BMSCs attached on titanium for evaluating the avidity bone to implant exhibited that there was no difference in the affinity of maxilla and iliac crest BMSCs to titanium. Titanium-attached maxilla BMSCs, however, were apparently more osteogenically responsive than iliac crest cells based on calcium accumulation and gene expression of alkaline phosphatase and osteopontin [37]. Akintoye et al. have also studied the skeletal site-specific osteogenic response of BMSC to BMP-2 stimulation [38]. They reported orofacial BMSC displayed high expression of osteogenic markers in response to BMP-2 in contrast to the low response of adult iliac crest BMSC. They also reported that mandible BMSCs were more susceptible to bisphosphonates than iliac crest BMSC [39]; orofacial BMSC survived higher radiation doses and recovered quicker than iliac crest BMSC [40]. Osteoclastogenic potential of jaw and long-bone-derived osteoclasts have different dynamics, and this might primarily due to differences in the cellular composition of the bone site-specific marrow [41]. Aghaloo et al. established a protocol for rat mandible and long-bone marrow stromal cell isolation and culture. Upon implantation into nude mice, mandible BMSCs formed 70% larger bone nodules containing three-fold more mineralized bone compared with long-bone BMSCs [42]. Alveolar BMSCs are obtained from older individuals, and the donor age has little effect on their gene expression pattern [43]. According to aforementioned studies, usage of alveolar BMSCs might have high advantages for alveolar bone regeneration compared with iliac BMSCs; however, establishing the protocol of harvesting BMSCs in low invasive way is still unclear.

## 4. Periosteal Tissue from Oral Site

Usage of periosteal cells from periosteum was originally reported by Breitbart et al. on rabbit experiments [44]. Outgrowthed periosteal cells were cultured with dexamethasone contained medium, and cell/polyglycolic acid non-woven fiver scaffolds complex showed significant bone formation on calvarial defect compared with scaffold only. Adult human periosteal cells from tibia include multipotent clonogenic cells [45], while, in the case of oral tissue, periosteal cells isolated from the mandibular angle of human with *β*-tricalcium phosphate granules have shown that combined treatment with bFGF and BMP-2 can make periosteal cells a highly useful source of bone regeneration [46]. In nude mouse subcutaneously model, acid-treated HA block cultured with human periosteal cells complex had significant osteogenic potential at the site of implantation *in vivo* [47], while in canine model for peri-implant bone regeneration, periosteum-derived cells in conjunction with e-PTFE membranes did not provide additional benefit [48]. Comparing with proliferated periosteal cells, Cicconetti et al. harvested marrow cells from maxillary tuberosity bone. They concluded that both periosteal cells and marrow stromal cells showed comparable phenotypic profiles and both cell populations formed bone upon ectopic *in vivo* transplantation [49]. Harvesting periosteal cells is relatively invasive treatment; for instance, it is still unclear the collection quantity for required bone regeneration and also periosteal tissue is hard to collect by general practitioners.

## 5. Gingival Tissue

During dental surgery, gingival tissue could be obtained frequently as a discarded biological sample. Wound healing within the gingiva is characterized by markedly reduced inflammation, scarless healing, rapid reepithelialization, contrary to the common scar formation present in skin [50, 51]. Recently, several reports have indicated the presence of progenitor cells in gingival connective tissue [52, 53]. Tang et al. reported gingival tissue contains tissue-specific mesenchymal stem cell population and is an ideal resource for immunoregulatory therapy, using human normal and hyperplastic gingival tissues [54]. The ratio of these progenitors in gingival fibroblasts, however, might be very low rather than bone marrow, periosteal tissue, or bone chips. Nevertheless, enrichment of progenitor cells that show characteristics with of differentiation as osteoblastic cells entail for usage in bone tissue engineering.

## 6. Conclusion

Bone marrow-derived cells, called mononuclear cells or marrow mesenchymal cells, are essentially different from osteoblastic cells derived from bone chip. In a case of small animal model, it is hard to separate mandibular bone marrow cells to bone lining osteoblastic cells [55]. In bulk, however, this bone/BMSCs possess unique stem cell properties that the size of alveolar bone has restrained the precise analysis of BMSCs phenotype. Furthermore, diversity of culture methods made us to confuse. As we use outgrowth cells from tissues on the other side, the population cultured cells is influenced by their migration ability. Treating tissue with enzyme to disperse cells from tissue, however, cell population is not dependent on migration ability. Thus, developing stable and universal methods to harvest and culture osteogenic cells for bone regeneration is prerequisite.
